# Improving the Clinical Diagnosis of Influenza—a Comparative Analysis of New Influenza A (H1N1) Cases

**DOI:** 10.1371/journal.pone.0008453

**Published:** 2009-12-29

**Authors:** Adrian K. Ong, Mark I. Chen, Li Lin, Adriana S. Tan, Ni Win Nwe, Timothy Barkham, Seow Yian Tay, Yee Sin Leo

**Affiliations:** 1 Department of Infectious Disease, Tan Tock Seng Hospital, Singapore; 2 Department of Emergency Medicine, Tan Tock Seng Hospital, Singapore; 3 Department of Laboratory Medicine, Tan Tock Seng Hospital, Singapore; Washington University School of Medicine, United States of America

## Abstract

**Background:**

The presentation of new influenza A(H1N1) is broad and evolving as it continues to affect different geographic locations and populations. To improve the accuracy of predicting influenza infection in an outpatient setting, we undertook a comparative analysis of H1N1(2009), seasonal influenza, and persons with acute respiratory illness (ARI) in an outpatient setting.

**Methodology/Principal Findings:**

Comparative analyses of one hundred non-matched cases each of PCR confirmed H1N1(2009), seasonal influenza, and ARI cases. Multivariate analysis was performed to look for predictors of influenza infection. Receiver operating characteristic curves were constructed for various combinations of clinical and laboratory case definitions. The initial clinical and laboratory features of H1N1(2009) and seasonal influenza were similar. Among ARI cases, fever, cough, headache, rhinorrhea, the absence of leukocytosis, and a normal chest radiograph positively predict for both PCR-confirmed H1N1-2009 and seasonal influenza infection. The sensitivity and specificity of current WHO and CDC influenza-like illness (ILI) criteria were modest in predicting influenza infection. However, the combination of WHO ILI criteria with the absence of leukocytosis greatly improved the accuracy of diagnosing H1N1(2009) and seasonal influenza (positive LR of 7.8 (95%CI 3.5–17.5) and 9.2 (95%CI 4.1–20.3) respectively).

**Conclusions/Significance:**

The clinical presentation of H1N1(2009) infection is largely indistinguishable from that of seasonal influenza. Among patients with acute respiratory illness, features such as a temperature greater than 38°C, rhinorrhea, a normal chest radiograph, and the absence of leukocytosis or significant gastrointestinal symptoms were all positively associated with H1N1(2009) and seasonal influenza infection. An enhanced ILI criteria that combines both a symptom complex with the absence of leukocytosis on testing can improve the accuracy of predicting both seasonal and H1N1-2009 influenza infection.

## Introduction

A novel influenza of swine origin, designated as new influenza A (H1N1), emerged in late April 2009. Since then, it has spread globally resulting in the first new pandemic of the 21^st^ century. The initial epidemiology and presentation of the disease has been notable for severe respiratory disease and mortality in persons less than 60 years and with co-morbidities [1], [2]. Its presentation is however broad and evolving as it continues to affect different geographic locations and populations.

From a health perspective, differentiating the new influenza A(H1N1) (hence referred to as H1N1(2009)) and influenza viruses from other pathogens presenting with symptoms of acute respiratory illness (ARI) is valuable. Making such a distinction serves to both improve individual case management—given the availability of safe and effective anti-influenza drugs to which H1N1(2009) remains presently largely susceptible to—and to augment wider public health surveillance and mitigation measures [1], [2], [3]. The clinical diagnosis of influenza infection is however often elusive given its non-specific presentation. The use of a simple symptom complex for influenza-like illnesses (ILI) at the primary care level can serve as convenient predictive tools for influenza infection, especially in the setting of an influenza community outbreak. However the sensitivity and positive predictive value of such symptom complexes or definitions vary widely depending on the prevalence of disease and population tested [4].

We undertook this study to better define the clinical and laboratory presentation of H1N1(2009) in an acute care outpatient setting. In addition, we sought to identify predictors of H1N1(2009) infection and validate the use of current clinical case definitions for influenza-like illness in predicting both H1N1(2009) and seasonal influenza.

## Methods

We conducted a retrospective review on the outpatient presentation of adult patients seen at the Tan Tock Seng Hospital (TTSH) for suspect influenza in the “containment period” of the H1N1(2009) epidemic in Singapore. TTSH is a 1500-bed tertiary hospital in Singapore and was designated the primary national center for adult influenza screening, treatment and isolation of H1N1(2009) cases. In line with enhanced surveillance and public health control measures during the containment period, the Ministry of Health instructed that all travelers returning from H1N1(2009) affected areas with ARI symptoms were to be referred to the hospital for screening and evaluation [5], [6]. Contacts of such patients were also to be referred to the hospital for H1N1(2009) screening. All patients had clinical samples collected at presentation for complete blood counts, serum chemistry and a chest radiograph (CXR). Two sets of nasal/throat swabs for influenza testing were collected from each patient. During the containment period, patients testing negative on influenza PCR were discharged if clinically stable, while all PCR-confirmed H1N1(2009) cases were admitted to the hospital for quarantine until negative nasal/throat viral shedding had been documented on serial PCR tests.

We selected our patients using a hospital influenza screening database of adult patients (age> = 16 years) seen during the epidemic containment period, which lasted from 27 Apr 2009 till end of June 2009. We included the first 100 consecutive adult cases of PCR-confirmed 2009-H1N1, and then used random number generators to sample one hundred non-matched cases of seasonal influenza and ARI adult patients. The patients presented between the following dates: H1N1(2009) cases between 26 May to 27 June 2009, seasonal influenza cases between 27 Apr to 11 June 2009 and ARI cases between 27 Apr to 11 June 2009. The choice of date ranges was influenced by our intent to obtain a sufficient number of patients from each group who were referred during the epidemic containment period while accounting for two key logistical limitations – that the first H1N1(2009) patient in Singapore was diagnosed only on 26 May 2009, and the decision to conserve laboratory testing policy after 11 June 2009 by testing ARI cases only to rule out H1N1(2009) rather than against the entire panel of seasonal influenza strains. ARI cases were defined as anyone who had self-reported influenza-like symptoms including chills/feverishness, cough, sore throat, headache, rhinorrhea, and/or myalgia, with or without a documented fever (body temperature >37.5°C. Clinical, epidemiological, radiology and laboratory data for each patient were extracted to a standardized clinical research form.

### Ethics Statement

An expedited review by the National Healthcare Group institutional review board of the hospital approved the study protocol (DSRB Domain E/09/344). A waiver of consent was obtained given the retrospective nature of the research and that the research involved no more than minimal risk to subjects nor involved any procedures for which written consent is normally required outside of the research context.

### Statistical Analyses

Statistical analyses were performed using STATA software (version 10, StataCorp, Texas). The Student's *t* test was used for comparison of continuous variable, and Fisher's exact test was used for comparison of dichotomous variables. For data that were not normally distributed, the Wilcoxon rank-sum test for continuous variables was used. Variables found to be statistically significant in uni-variate analyses were entered into multivariate analysis using a logistic regression model to identify independent risk factors for being H1N1(2009) positive. A two-tailed *p* value of <0.05 was considered statistically significant.

We compared the performance of individual and combinations of variables for the diagnosis of both H1N1(2009) and seasonal influenza by using the RT-PCR as the reference standard. We calculated the sensitivity, specificity, positive predictive value (PPV), negative predictive value (NPV), likelihood ratio (LR) for a positive test (LRP), and LR for a negative test (LRN) with 95 percent confidence intervals (CI). LRs indicate by how much a given diagnostic test result will raise or lower the pretest probability of the target disorder. LRPs of >10 or LRNs of <0.1 generate large and often conclusive changes from pretest probability to posttest probability, LRPs of 5 to 10 and LRNs of 0.1 to 0.2 generate moderate shifts in pretest probability to posttest probability, LRPs of 2 to 5 and LRNs of 0.2 to 0.5 generate small (but sometimes important) changes in probability, and LRPs of 1 to 2 and LRNs of 0.5 to 1 alter probability to a small (and rarely important) degree [7]. Receiver operating characteristic curves were plotted for various combinations of clinical and laboratory case definitions. The diagnostic accuracy of each parameter and symptom combination was assessed by calculating its area under receiver operating characteristic curves (AUROC). AUROC analysis was used to answer the question of how accurate combinations of symptoms, signs and investigations are in identifying H1N1(2009) and seasonal influenza diseased patients.

### Laboratory Confirmation

Combined nasal and throat flocked swabs (Copan, Italy) in RT-UTM (Copan, Italy) were vortexed and nucleic acids extracted with a NucliSENS® easyMAG® instrument (Biomerieux) or with the EZ1 virus minikit v2.0 on an EZ1 Advanced XL (Qiagen). Reverse transcription PCR (RT-PCR) was migrated across three platforms as the pandemic progressed; a multiplex in-house gel based method (100% sensitivity and specificity for detection and subtyping compared with ResplexII, Qiagen, Germany), a Taqman based real time in-house singleplex assay specific to H1N1 (2009) run on a Lightcycler (Roche, Germany) and an in-house Taqman based real time multiplex assay including H1N1 (2009) specific reagents run on a Stratagene Mx3005P (Agilent, USA), (all three assays' manuscript in preparation for publication). Cases of Influenza A H1N1 were confirmed as either seasonal H1N1 or as H1N1 (2009) by the National Public Health Laboratory, with the use of two RT-PCR assays including the CDC method, sequencing of the matrix gene and viral culture on Madin-Darby canine kidney cells with subsequent typing with a DFA kit (LIGHT DIAGNOSTICS™, Millipore) and subtyping with RT-PCR.

## Results

**Figure 1:**
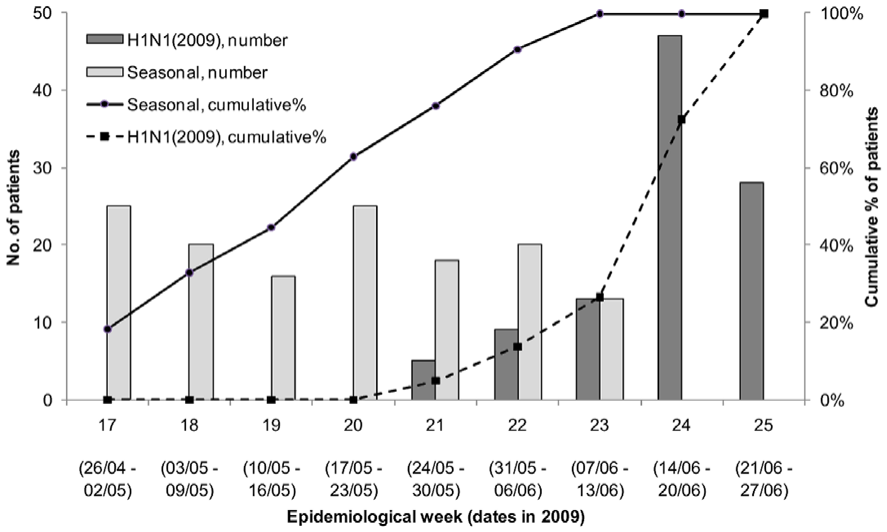
Date of presentation of adult H1N1(2009) and seasonal influenza cases by epidemiological week. Note that there was inconsistent testing for influenza strains other than H1N1(2009) after 11 June 2009 (Week 23), and the data for seasonal influenza is hence censored after week 23.

Between 27 April and 11 June, a total of 983 patients presented to the Emergency Department to be screened for influenzaThis included 137 adults with PCR-confirmed seasonal influenza strains presenting between the above dates (Figure 1), from which 100 patients (83 with influenza A(H3N2), 7 seasonal influenza A(H1N1) and 10 cases of influenza B were randomly selected. There were also 20 patients with PCR-confirmed H1N1(2009) as of 11 June 2009, and additional cases of H1N1(2009) up to 27 June 2009 were included to make up 100 subjects for analyses. Finally, 100 patients negative for all influenza strains were randomly selected from the remaining influenza negative ARI patients (presenting between 27 April and 11 June 2009). Demographic information, underlying co-morbid conditions and travel history of the selected patients from all three comparative groups are presented in Table 1. The majority of the 100 laboratory-confirmed cases of H1N1(2009) presenting to the emergency department were adults younger than 30 years of age. The H1N1(2009) individuals were less likely to belong to a major ethnic group of Singapore and less likely to have a co-morbid condition. In of all three groups of patients, more than three quarters reported recent travel outside of Singapore.

**Table 1:** Demographics, co-morbidities, and history of travel.

Patient characteristics		A: H1N1 (2009)(N = 100)	B: Other influenza strains(N = 100)	C: No influenza on PCR(N = 100)	*p*-Value A vs B	A vs C	B vs C
**Age in years**	Mean	27.2	37.6	37.3	<0.001*	<0.001*	
	Range	(16–56)	(18–75)	(20–94)			
**Age distribution, %**					<0.001†	<0.001†	
	<30 years	74%	26%	36%			
	30 to 49 years	21%	60%	44%			
	≥50 years	5%	14%	20%			
**Male gender, %**		53%	59%	47%			
***Ethnicity, %***						0.024†	
	Chinese	45%	59%	64%			
	Malay	9%	9%	5%			
	Indian	7%	6%	9%			
	Others	39%	26%	22%			
***Comorbid conditions, %***							
	Diabetes	0%	2%	5%			
	Hypertension/dyslipidemia	2%	9%	10%		0.033†	
	Asthma/bronchitis/COPD	5%	7%	8%			
	Childhood asthma	3%	3%	4%			
	Cardiovascular disease	0%	1%	4%			
	Immuno-compromised state	1%	2%	0%			
	Others	7%	10%	10%			
***Risk factors for severe influenza infection, %***							
	Age ≥65 years	0%	5%	4%			
	Significant co-morbidities‡	6%	10%	14%			
	Significant co-morbidities or age ≥65‡	6%	12%	16%		0.024†	
**Reported travel history, %**		79%	74%	93%		0.004†	<0.001†

* 2-sided p-value by Student's t test.

† 2-sided p-value by chi-squared test or Fisher's exact test

‡ Includes diabetes, asthma (excluding childhood asthma), COPD (chronic obstructive pulmonary disease), chronic bronchitis, cardiovascular disease (excluding hypertension) and conditions possibly causing an immuno-compromised state

More than half of the influenza cases presented within by day 3 of illness. Self-reported feverishness was a prominent presenting feature in individuals with either H1N1(2009) or seasonal influenza (Table 2). Cough, sore throat and rhinorrhea at presentation were common across all 3 groups but not myalgia and headache; only a few had lower respiratory tract symptoms such as dyspnea. Of patients with confirmed H1N1(2009) and seasonal influenza, 13% and 8% respectively had leukocytosis, whereas leukocytosis was found in approximately half of the non-influenza cases. Lymphopenia occurred less frequently in individuals with H1N1(2009) than in those with seasonal influenza (33% vs. 55%, p = 0.002) but more frequently than those without influenza (33% vs. 16%, p = 0.005). Abnormal chest radiographic findings were not common in any of the groups.

**Table 2:** Symptoms, clinical, laboratory, and radiological findings at presentation.

Patient characteristics		A: H1N1 (2009) (N = 100)	B: Other influenza strains (N = 100)	C: No influenza on PCR (N = 100)	*p*-Value		
					A vs B	A vs C	B vs C
**Day of illness at presentation, %**							
	Day 1	9%	7%	7%			
	Day 2	22%	22%	24%			
	Day 3	24%	29%	17%			
	Day 4 or later	45%	42%	52%			
**Constitutional symptoms, %**							
	Self-reported feverishness/chills/rigors	88%	92%	53%		<0.001†	<0.001†
	Myalgia	14%	23%	12%			<0.041†
	Lethargy/malaise	4%	2%	5%			
	Nausea/vomitting/anorexia	2%	7%	14%		0.003	
	Headache	19%	15%	10%			
**Respiratory symptoms, %**							
	Cough	80%	84%	63%		0.008†	0.001†
	Sore throat	51%	61%	58%			
	Rhinorrhea/nasal congestion	60%	59%	44%		0.024†	0.034†
	Lower respiratory tract symptoms	1%	6%	6%			
**Other symptoms, %**							
	Diarrhoea	4%	0%	9%			0.003†
	Abdominal discomfort	3%	2%	6%			
**Heart rate**						<0.001*	<0.001*
	Mean	101.0	104.5	94.0			
	Range	(70.0–196.0)	(75.0–143.0)	(63.0–128.0)			
**Body temperature**						<0.001*	<0.001*
	Mean	38.2	38.3	37.3			
	Range	(36.5–39.7)	(36.3–40.3)	(36.0–40.1)			
**Body temperature, %**						<0.001†	<0.001†
	T≤37.4°C	24%	18%	60%			
	T = 37.5°C to 37.7°C	13%	8%	11%			
	T = 37.8°C to 38.0°C	6%	11%	12%			
	T≥38.1°C	57%	63%	17%			
**Leucocytosis (leucocytes >9.3×10̂9/L)**		13%	8%	43%		<0.001†	<0.001†
**Lymphopenia (lymphocytes <0.9×10̂9/L)**		33%	55%	16%	0.002†	0.005†	<0.001†

*2-sided p-value by Student's t test.

† 2-sided p-value by chi-squared test or Fisher's exact test; only values significant at p<0.05 are shown.

**Figure 2:**
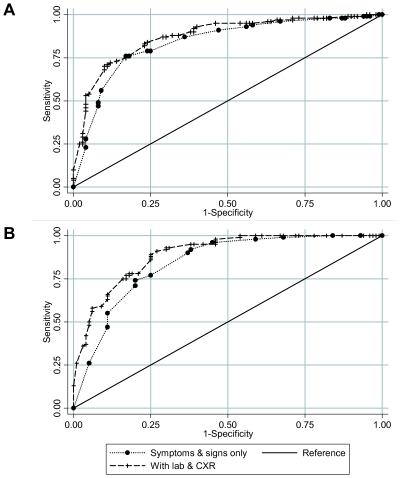
Receiver operating characteristic curves. (A) H1N1 versus acute respiratory illness, where Area under the receiver operating characteristic curve (AUROC) values are 0.839 for multivariate logistic regression model using symptoms and signs only, and 0.874 when adding laboratory and chest radiograph (CXR) findings. (B) seasonal influenza versus acute respiratory illness. AUROC values are 0.842 for symptoms and signs only, and 0.893 when adding laboratory and CXR findings.

On univariate analysis (Table 3), no single symptom, sign or laboratory feature was strongly predictive of H1N1(2009). The highest temperature threshold of 38.1°C or greater was most strongly predictive of influenza (crude odds ratio (OR) 6.45, 95%CI 3.2–13.4) while nausea, vomiting and anorexia was inversely associated with influenza (crude OR 0.1, 95%CI 0.0–0.6). On multivariate analysis using both clinical and laboratory predictors, H1N1(2009) patients were more likely to report feverishness, chills or rigors (Adjusted OR (aOR) = 3.8, 95%CI, 1.6–9.0); rhinorrhea or nasal congestion (aOR = 2.5, 95%CI, 1.2–5.3), and more likely to have a temperature of 38.1°C or higher (aOR = 6.7, 95%CI, 2.7–16.7) compared to patients with ARI. The absence of leukocytosis and a normal CXR increased the odds of having H1N1(2009) by greater than 4 and 6 fold respectively. Nausea, vomiting or anorexia negatively predicted for H1N1(2009) infection (aOR = 0.1, 95%CI, 0–0.3). In a separate multivariate analysis, the same clinical and laboratory features identified as significant for H1N1(2009) diagnosis were mostly found to be similarly important in predicting seasonal influenza (Table 4). Key differences were that several symptoms such as headache, nausea, vomiting or anorexia were not found to be significant when comparing seasonal influenza with ARI. However, higher odds ratios were observed for self-reported feverishness and cough, and lymphopenia was found to be a significant discriminator for seasonal influenza. In multivariate analysis, adding basic laboratory features and CXR findings marginally but significantly improved our ability to discriminate both H1N1(2009) and seasonal influenza from ARI (p = 0.03 and p = 0.006 respectively), as illustrated by the AUROC plots in Figure 2.

**Table 3:** Univariate and multivariate analyses comparing H1N1(2009) and ARI patients.

Patient characteristics		Univariate analysis			Multivariate analysis, symptoms only			Multivariate analysis with lab & CXR		
		Crude OR	(95% CI)	*p*-Value	Adjusted OR	(95% CI)	*p*-Value	Adjusted OR	(95% CI)	*p*-Value
**Constitutional symptoms, %**	Self-reported feverishness/chills/rigors	6.5	(3.2–13.4)	<0.001	4.0	(1.8–9.0)	0.001	3.8	(1.6–9.0)	0.003
	Headache	2.1	(0.9–4.8)	0.075		-		2.8	(0.9–9.1)	0.083
	Myalgia	1.2	(0.5–2.7)	0.674		-			-	
	Lethargy/malaise	0.8	(0.2–3.0)	0.734		-			-	
	Nausea/vomitting/anorexia	0.1	(0.0–0.6)	0.007	0.1	(0.0–0.4)	0.002	0.1	(0.0–0.3)	0.002
**Respiratory symptoms, %**	Cough	2.3	(1.2–4.4)	0.009	2.0	(0.9–4.3)	0.073	2.2	(1.0–5.0)	0.057
	Sorethroat	0.8	(0.4–1.3)	0.321		-			-	
	Rhinorrhea/nasal congestion	1.9	(1.1–3.3)	0.024	2.5	(1.3–5.1)	0.009	2.5	(1.2–5.3)	0.018
**Body temperature cut-off points**	T≥38.1°C	6.5	(3.4–12.5)	<0.001	6.4	(2.8–14.4)	<0.001	6.7	(2.7–16.7)	<0.001
	T≥37.8°C	4.2	(2.3–7.5)	<0.001		-			-	
	T≥37.5°C	4.8	(2.6–8.7)	<0.001		-			-	
**Laboratory and radiological findings**	Absence of leucocytosis (leucocytes >9.3×10̂9/L)	5.0	(2.5–10.2)	<0.001		-		4.5	(1.9–10.7)	0.001
	Lymphopenia (lymphocytes <0.9×10̂9/L)	2.6	(1.3–5.1)	0.006		-			-	
	Normal chest radiograph	3.3	(1.0–10.5)	0.047		-		6.2	(1.2–30.6)	0.026

**Table 4:** Univariate and multivariate analyses comparing seasonal influenza and ARI patients.

Patient characteristics		Univariate analysis			Multivariate analysis, symptoms only			Multivariate analysis with lab & CXR		
		Crude OR	(95% CI)	*p*-Value	Adjusted OR	(95% CI)	*p*-Value	Adjusted OR	(95% CI)	*p*-Value
**Constitutional symptoms, %**	Self-reported feverishness/chills/rigors	10.2	(4.5–23.2)	<0.001	7.7	(3.1–19.2)	<0.001	5.6	(2.0–15.9)	0.001
	Headache	1.6	(0.7–3.7)	0.288		-			-	
	Myalgia	2.2	(1–4.7)	0.044		-			-	
	Lethargy/malaise	0.4	(0.07–2.0)	0.264		-			-	
	Nausea/vomitting/anorexia	0.5	(0.2–1.2)	0.113		-			-	
**Respiratory symptoms, %**	Cough	3.1	(1.6–6.0)	0.001	3.7	(1.7–8.5)	0.002	4.0	(1.6–10.3)	0.003
	Sorethroat	1.1	(0.6–2.0)	0.666		-			-	
	Rhinorrhea/nasal congestion	1.8	(1.0–3.2)	0.034	2.4	(1.2–5.0)	0.014	2.3	(1.0–5.0)	0.040
**Body temperature cut-off points**	T≥38.1°C	8.3	(4.3–16.1)	<0.001	5.8	(2.8–12.1)	<0.001	4.8	(2.0–11.7)	0.001
	T≥37.8°C	7.0	(3.7–13.0)	<0.001		-			-	
	T≥37.5°C	6.8	(3.6–13.1)	<0.001		-			-	
**Laboratory and radiological findings**	Absence of leucocytosis (leucocytes >9.3×10̂9/L)	8.7	(3.8–19.8)	<0.001		-		8.5	(3.0–23.7)	<0.001
	Lymphopenia (lymphocytes <0.9×10̂9/L)	6.4	(3.3–12.5)	<0.001		-		3.1	(1.2–7.7)	0.018
	Normal chest radiograph	2.6	(0.9–7.7)	0.085		-		3.6	(0.8–15.9)	0.088

Table 5 gives the likelihood ratios for key variables significantly associated with influenza infection. Body temperature of 38.1°C or more was the single variable best able to distinguish ARIs, but the LRP and LRN values indicate that it could at best generate small changes in posttest probabilities. Table 5 also presents results for combinations of symptoms and laboratory criteria.

**Table 5:** Sensitivity and specificity of combinations of symptoms, signs, and investigations for distinguishing H1N1(2009).

Symptoms and signs		H1N1(2009) vs ARI					Seasonal influenza vs ARI				
		Sensitivity	Specificity	AUROC*	Positive LR† (95% CI)	Negative LR† (95% CI)	Sensitivity	Specificity	AUROC*	Positive LR† (95% CI)	Negative LR† (95% CI)
**Single symptom or sign**											
	Self-reported feverishness/chills/rigors	88%	47%	68%	1.7 (1.4–2.0)	0.3 (0.1–0.5)	92%	47%	70%	1.7 (1.4–2.1)	0.2 (0.1–0.3)
	Headache	19%	90%	55%	1.9 (0.9–3.9)	0.9 (0.8–1.0)	15%	90%	53%	1.5 (0.7–3.2)	0.9 (0.9–1.0)
	Absence of nausea/vomiting/loss of appetite	98%	14%	56%	1.1 (1.0–1.2)	0.1 (0.0–0.6)	93%	14%	54%	1.1 (1.0–1.2)	0.5 (0.2–1.2)
	Cough	80%	37%	59%	1.3 (1.1–1.5)	0.5 (0.3–0.9)	84%	37%	61%	1.3 (1.1–1.6)	0.4 (0.3–0.7)
	Rhinorrhoea/nasal congestion	60%	56%	58%	1.4 (1.0–1.8)	0.7 (0.5–1.0)	59%	56%	58%	1.3 (1.0–1.8)	0.7 (0.5–1.0)
**Body temperature cut-off points**											
	T≥38.1°C	57%	83%	70%	3.4 (2.1–5.3)	0.5 (0.4–0.7)	63%	83%	73%	3.7 (2.3–5.9)	0.4 (0.3–0.6)
	T≥37.8°C	63%	71%	67%	2.2 (1.5–3.1)	0.5 (0.4–0.7)	74%	71%	73%	2.6 (1.8–3.5)	0.4 (0.3–0.5)
	T≥37.5°C	76%	60%	68%	1.9 (1.5–2.5)	0.4 (0.3–0.6)	82%	60%	71%	2.1 (1.6–2.7)	0.3 (0.2–0.5)
**Laboratory findings**											
	Absence of leucocytosis (leucocytes >9.3×10̂9/L)	87%	43%	65%	1.5 (1.3–1.8)	0.3 (0.2–0.5)	92%	43%	68%	1.6 (1.3–1.9)	0.2 (0.1–0.4)
	Lymphopenia (lymphocytes <0.9×10̂9/L)	33%	84%	59%	2.1 (1.2–3.5)	0.8 (0.7–0.9)	55%	84%	70%	3.4 (2.1–5.6)	0.5 (0.4–0.7)
	Normal chest radiograph	96%	12%	54%	1.1 (1.0–1.2)	0.3 (0.1–1.0)	95%	12%	54%	1.1 (1.0–1.2)	0.4 (0.2–1.1)
**Selected combinations of symptoms and signs**											
	T≥38.1°C AND [cough OR sorethroat]: WHO ILI criteria	50%	87%	69%	3.8 (2.2–6.6)	0.6 (0.5–0.7)	59%	87%	73%	4.5 (2.7–7.7)	0.5 (0.4–0.6)
	T≥37.8°C AND [cough OR sorethroat]: CDC ILI criteria	55%	78%	67%	2.5 (1.7–3.8)	0.6 (0.5–0.7)	69%	78%	74%	3.1 (2.1–4.6)	0.4 (0.3–0.5)
	T≥37.5°C AND [cough OR sorethroat]	67%	67%	67%	2.0 (1.5–2.8)	0.5 (0.4–0.7)	76%	67%	72%	2.3 (1.7–3.1)	0.4 (0.2–0.5)
	self-reported feverishness/chills/rigors AND [cough OR sorethroat]	78%	58%	68%	1.9 (1.4–2.4)	0.4 (0.3–0.6)	86%	58%	72%	2.0 (1.6–2.6)	0.2 (0.1–0.4)
	T≥38.1°C AND [cough OR rhinorrhoea/nasal congestion]	50%	88%	69%	4.2 (2.4–7.3)	0.6 (0.5–0.7)	60%	88%	74%	5.0 (2.9–8.7)	0.5 (0.4–0.6)
	T≥37.8°C AND [cough OR rhinorrhoea/nasal congestion]	55%	85%	70%	3.7 (2.2–6.0)	0.5 (0.4–0.7)	71%	85%	78%	4.7 (2.9–7.7)	0.3 (0.2–0.5)
	T≥37.5°C AND [cough OR rhinorrhoea/nasal congestion]	67%	75%	71%	2.7 (1.9–3.9)	0.4 (0.3–0.6)	78%	75%	77%	3.1 (2.2–4.5)	0.3 (0.2–0.4)
	Self-reported feverishness/chills/rigors AND [cough OR rhinorrhoea/nasal congestion]	82%	61%	70%	2.1 (1.6–2.7)	0.3 (0.2–0.5)	88%	61%	75%	2.3 (1.7–2.9)	0.2 (0.1–0.3)
**Combinations of symptoms & signs with CBC and CXR results**											
	WHO ILI criteria AND absence of leucocytosis	47%	94%	71%	7.8 (3.5–17.5)	0.6 (0.5–0.7)	55%	94%	75%	9.2 (4.1–20.3)	0.5 (0.4–0.6)
	WHO ILI criteria AND lymphopenia	24%	93%	59%	3.4 (1.5–7.6)	0.8 (0.7–0.9)	37%	93%	65%	5.3 (2.5–11.3)	0.7 (0.6–0.8)
	WHO ILI criteria AND normal chest radiograph	48%	91%	70%	5.3 (2.8–10.3)	0.6 (0.5–0.7)	56%	91%	74%	6.2 (3.3–11.9)	0.5 (0.4–0.6)
	CDC ILI criteria AND absence of leucocytosis	50%	90%	70%	5.0 (2.7–9.3)	0.6 (0.5–0.7)	65%	90%	78%	6.5 (3.5–11.9)	0.4 (0.3–0.5)
	CDC ILI criteria AND lymphopenia	24%	91%	58%	2.7 (1.3–5.4)	0.8 (0.7–0.9)	42%	91%	67%	4.7 (2.4–9.1)	0.6 (0.5–0.8)
	CDC ILI criteria AND normal chest radiograph	53%	83%	68%	3.1 (1.9–5.0)	0.6 (0.5–0.7)	65%	83%	74%	3.8 (2.4–6.0)	0.4 (0.3–0.6)
	Self-reported feverishness/chills/rigors AND [cough OR rhinorrhoea/nasal congestion] AND absence of leucocytosis	71%	80%	76%	3.6 (2.4–5.4)	0.4 (0.3–0.5)	81%	80%	81%	4.1 (2.7–6.1)	0.2 (0.2–0.4)

*AUROC: Area under Receiver Operating Characteristic curve.

† LR: likelihood ratio.

Forty percent of all H1N1 participants presented with symptoms that met the WHO's criteria for influenza-like illness, i.e. presence of fever (temperature >38.0C) and an upper respiratory symptoms such as cough or sore throat. These findings in combination generated a sensitivity of 50% and specificity of 87% in differentiating H1N1(2009) from non-influenza illnesses. The positive LR of both current WHO and CDC ILI criteria were modest in aiding H1N1(2009) diagnosis (3.8 and 2.5 respectively) and seasonal influenza (4.5 and 3.1 respectively). By AUROC analyses, the addition of complete blood count criteria and chest radiograph results to a combination of clinical predictors allowed for greater diagnostic accuracy. The absence of leukocytosis (defined as leucocytes <9.3×10̂9/L) in particular, was the best laboratory criteria that improved the specificity and positive LR of present WHO and CDC ILI criteria. The combination of WHO ILI criteria with the absence of leukocytosis had a positive LR of 7.8 (95%CI 3.5–17.5) in diagnosing H1N1(2009) and 9.2 (95%CI 4.1–20.3) for seasonal influenza.

## Discussion

The clinical spectrum of H1N1(2009) is still being defined in different populations and clinical settings. Although the clinical features of patients with influenza during outbreaks have previously been described [8], [9], data on the differences in clinical presentation between H1N1(2009), seasonal influenza and ARIs are sparse. Previous reports of influenza have noted clinical dissimilarities in the observed presentation and course of influenza infection between various influenza subtypes [10], [11], [12].

This study provides comparative evidence that the initial presentation of novel H1N1(2009) in adults does not differ significantly from that of contemporary seasonal influenza—the majority (83 percent) of which were influenza A (H3N2). With the exception of lymphopenia, the presenting symptoms, clinical findings, radiographic and basic laboratory results of the two infections were largely similar. Our findings on H1N1(2009) are both reassuring and mostly consistent with information published to date [1], [2], [13], [14]. In our series however, gastrointestinal (GI) symptoms did not feature prominently among H1N1(2009) cases; this is in contrast to earlier clinical observations where GI symptoms have been noted in a high percentage (25 to 38 percent) of cases and findings in animal models suggesting greater viral tropism for the GI tract [1], [2], [15]. Also lymphopenia was not as common a finding in H1N1(2009) infections as compared to seasonal influenza. This could be attributable to the greater degree of lymphopenia previously described in A(H3N2) infections [10], a subtype that comprised the major putative agent in our seasonal influenza group.

Beyond the clinical presentation, our study also explored the accuracy of various clinical predictors for the diagnosis of H1N1(2009) in a population of adult patients with acute respiratory illness. No single clinical finding had a positive LR high enough to discriminate for H1N1(2009) nor a negative LR low enough to exclude it. On multivariate analysis, a fever greater than 38°C, feverishness (chills, rigors, or self-reported feverishness), rhinorrhea, a normal chest radiograph and the absence of leukocytosis and significant gastrointestinal symptoms were all positively associated with H1N1(2009) infection. Most of the factors which predicted H1N1(2009) infection in comparison with acute respiratory illnesses were also found to be relevant for seasonal influenza. Again, no single clinical finding had sufficient high positive LR or low negative LR, but with the WHO ILI criteria in combination with the absence of leukocytosis gave reasonably high LRP for seasonal influenza.

It is appreciated that the sensitivity of clinical predictors for influenza varies depending on a multitude of factors including the prevalence of disease, age, underlying illnesses, duration of symptoms prior to consultation, and the vaccination rate in the population tested. Published data to date have shown varying positive predictive values with the use of fever and cough as clinical predictors, ranging from 35 to 83 percent [4], [8], [16], [17]. Most of these studies have been conducted in the setting of community outbreaks of seasonal influenza. In our analysis, current CDC and WHO criteria for ILI are both fairly specific for H1N1(2009) but have only modest positive LRs in our influenza PCR-confirmed population. The WHO criterion with a higher temperature cut-off was predictably more specific and accurate in ruling in a diagnosis of H1N1(2009) than present CDC ILI criteria. The addition of routine laboratory test results such as a complete blood count or chest radiographs expectedly increases the specificity and positive predictive value of these ILI definitions. In particular, the absence of leukocytosis added the greatest discriminatory power when combined with the WHO ILI definition. The use of such a modified ILI definition would be of special practical benefit in settings that screen for influenza (including H1N1(2009)) and have facilities for basic hematological testing, such as emergency rooms and primary care facilities. Although influenza can be diagnosed with commercially available rapid test kits, the sensitivity of such test kits have been shown to be low in some reports [18], [19], [20], [21], [22], [23]. Augmenting current clinical criteria with additional laboratory criteria—specifically the absence of leukocytosis—will allow for greater accuracy in distinguishing influenza from other acute respiratory illnesses and permit the earlier initiation of appropriate therapy and public health measures.

The limitations of our study include the lack of evaluative vaccination history data and the retrospective nature of our data collection. Also, our analyzed population comprised mainly outpatient young adults thereby precluding the extrapolation our results to pediatric patients and those in special settings.

Our analysis demonstrates that the clinical presentation of H1N1(2009) infection is largely indistinguishable from that of seasonal influenza. However among patients with acute respiratory illness, features such as a temperature greater than 38°C, rhinorrhea, a normal chest radiograph, and the absence of leukocytosis or significant gastrointestinal symptoms were all positively associated with H1N1(2009) and seasonal influenza infection. The use of enhanced ILI criteria in certain settings, that combine both a symptom complex with the absence of leukocytosis on basic laboratory testing, can considerably improve the accuracy of establishing the diagnosis of influenza.
